# Marine Hospitals on the Mississippi

**Published:** 1834

**Authors:** 


### IV. National Marine Hospitals on the Mississippi and its Tributary Streams.

Dr. Cornelius Campbell, a respectable physician and public spirited citizen of St. Louis, has been for some time engaged in arousing the attention of his friends and acquaintances in the West, to the great importance of such establishments. Our limits in this number, do not permit our presenting to the profession in the Valley of the Mississippi, even an outline of his views; but we propose to make them the subject of an article in the next number. Meanwhile we hope that every reader, will give to the matter that consideration which its importance obviously demands, and be prepared to lend his aid, in an application to the General Government, whenever the time for making it may arrive.

